# Supplementary material for: Modeling glioblastoma heterogeneity as a dynamic network of cell states
Source: Mol Syst Biol. 2021 Sep 16;17(9):e10105. doi: 10.15252/msb.202010105 (PMC8444284; doi:10.15252/msb.202010105)
Supplement: Supplementary file 6 — Source Data for Figure 5 [file MSB-17-e10105-s004.zip › Figure5A_sourcedata/GSEA_3071/nosigdb_stateI.GseaPreranked.1622795145664/DOROSZKO_U3054MG_CLUSTER_4.html]

Details for gene set DOROSZKO\_U3054MG\_CLUSTER\_4[GSEA]

|  || Dataset | state13071 |
| Phenotype | NoPhenotypeAvailable |
| Upregulated in class | na\_neg |
| GeneSet | DOROSZKO\_U3054MG\_CLUSTER\_4 |
| Enrichment Score (ES) | -0.55547196 |
| Normalized Enrichment Score (NES) | -3.5163367 |
| Nominal p-value | 0.0 |
| FDR q-value | 0.0 |
| FWER p-Value | 0.0 |
Table: GSEA Results Summary

  

Fig 1: Enrichment plot: DOROSZKO\_U3054MG\_CLUSTER\_4      
 Profile of the Running ES Score & Positions of GeneSet Members on the Rank Ordered List

  

| PROBE | GENE SYMBOL | GENE\_TITLE | RANK IN GENE LIST | RANK METRIC SCORE | RUNNING ES | CORE ENRICHMENT || 1 | SLC20A1 |  |  | 116 | 1.035 | -0.0329 | No |
| 2 | SLC1A5 |  |  | 199 | 0.831 | -0.0558 | No |
| 3 | SOD3 |  |  | 219 | 0.786 | -0.0585 | No |
| 4 | PHGDH |  |  | 279 | 0.699 | -0.0745 | No |
| 5 | NTM |  |  | 427 | 0.572 | -0.1194 | No |
| 6 | PSMB9 |  |  | 437 | 0.560 | -0.1199 | No |
| 7 | TMEM243 |  |  | 530 | 0.490 | -0.1474 | No |
| 8 | MTHFD2 |  |  | 588 | 0.459 | -0.1638 | No |
| 9 | SHMT2 |  |  | 777 | 0.390 | -0.2228 | No |
| 10 | DUSP23 |  |  | 786 | 0.387 | -0.2237 | No |
| 11 | JPT2 |  |  | 1037 | 0.321 | -0.3029 | No |
| 12 | IDH2 |  |  | 1324 | 0.264 | -0.3939 | No |
| 13 | NPW |  |  | 1372 | 0.256 | -0.4080 | No |
| 14 | CCDC34 |  |  | 1376 | 0.255 | -0.4078 | No |
| 15 | MSH2 |  |  | 1438 | -0.252 | -0.4264 | No |
| 16 | CEP152 |  |  | 1446 | -0.253 | -0.4276 | No |
| 17 | STIP1 |  |  | 1453 | -0.253 | -0.4284 | No |
| 18 | RAD1 |  |  | 1475 | -0.255 | -0.4341 | No |
| 19 | CCND3 |  |  | 1482 | -0.255 | -0.4349 | No |
| 20 | SRSF10 |  |  | 1489 | -0.256 | -0.4358 | No |
| 21 | VOPP1 |  |  | 1490 | -0.256 | -0.4347 | No |
| 22 | NUP85 |  |  | 1492 | -0.256 | -0.4339 | No |
| 23 | ZGRF1 |  |  | 1505 | -0.258 | -0.4366 | No |
| 24 | C1orf35 |  |  | 1512 | -0.258 | -0.4374 | No |
| 25 | NR2C2AP |  |  | 1527 | -0.260 | -0.4408 | No |
| 26 | CTPS1 |  |  | 1540 | -0.261 | -0.4436 | No |
| 27 | RBBP4 |  |  | 1549 | -0.262 | -0.4450 | No |
| 28 | CHAF1B |  |  | 1554 | -0.262 | -0.4452 | No |
| 29 | TMEM54 |  |  | 1555 | -0.262 | -0.4440 | No |
| 30 | POLE2 |  |  | 1560 | -0.263 | -0.4442 | No |
| 31 | ARL6IP6 |  |  | 1577 | -0.265 | -0.4482 | No |
| 32 | SUPT16H |  |  | 1588 | -0.266 | -0.4503 | No |
| 33 | ALDH7A1 |  |  | 1615 | -0.269 | -0.4575 | No |
| 34 | MPHOSPH9 |  |  | 1623 | -0.270 | -0.4586 | No |
| 35 | HDGFL3 |  |  | 1626 | -0.270 | -0.4581 | No |
| 36 | IPO9 |  |  | 1634 | -0.272 | -0.4592 | No |
| 37 | SAE1 |  |  | 1638 | -0.273 | -0.4589 | No |
| 38 | SMC3 |  |  | 1650 | -0.274 | -0.4613 | No |
| 39 | PPAT |  |  | 1655 | -0.275 | -0.4614 | No |
| 40 | SNX5 |  |  | 1663 | -0.275 | -0.4625 | No |
| 41 | BUB3 |  |  | 1687 | -0.278 | -0.4687 | No |
| 42 | NUP210 |  |  | 1695 | -0.279 | -0.4697 | No |
| 43 | TEDC1 |  |  | 1698 | -0.279 | -0.4692 | No |
| 44 | RAD54L |  |  | 1702 | -0.280 | -0.4689 | No |
| 45 | ACD |  |  | 1713 | -0.282 | -0.4710 | No |
| 46 | RPA1 |  |  | 1717 | -0.282 | -0.4707 | No |
| 47 | NXT1 |  |  | 1722 | -0.283 | -0.4708 | No |
| 48 | PEG10 |  |  | 1734 | -0.284 | -0.4731 | No |
| 49 | KIF2C |  |  | 1735 | -0.284 | -0.4719 | No |
| 50 | PSMC3 |  |  | 1752 | -0.287 | -0.4758 | No |
| 51 | CCNB2 |  |  | 1757 | -0.288 | -0.4758 | No |
| 52 | MIS18BP1 |  |  | 1764 | -0.289 | -0.4765 | No |
| 53 | CENPJ |  |  | 1768 | -0.290 | -0.4762 | No |
| 54 | EXOSC9 |  |  | 1777 | -0.291 | -0.4775 | No |
| 55 | MPHOSPH6 |  |  | 1778 | -0.291 | -0.4763 | No |
| 56 | TRIM28 |  |  | 1789 | -0.292 | -0.4783 | No |
| 57 | USP37 |  |  | 1799 | -0.294 | -0.4799 | No |
| 58 | CASP8AP2 |  |  | 1857 | -0.300 | -0.4970 | No |
| 59 | CENPQ |  |  | 1860 | -0.300 | -0.4963 | No |
| 60 | TTF2 |  |  | 1873 | -0.301 | -0.4989 | No |
| 61 | ERCC6L |  |  | 1892 | -0.304 | -0.5034 | No |
| 62 | MTCH2 |  |  | 1924 | -0.308 | -0.5120 | No |
| 63 | PRPF4 |  |  | 1936 | -0.311 | -0.5142 | No |
| 64 | TIPIN |  |  | 1942 | -0.312 | -0.5145 | No |
| 65 | KPNA2 |  |  | 1944 | -0.312 | -0.5135 | No |
| 66 | CDC25A |  |  | 1953 | -0.313 | -0.5147 | No |
| 67 | SPDL1 |  |  | 1968 | -0.314 | -0.5179 | No |
| 68 | SPATA33 |  |  | 1972 | -0.314 | -0.5175 | No |
| 69 | SNRPA1 |  |  | 1984 | -0.316 | -0.5197 | No |
| 70 | VBP1 |  |  | 1993 | -0.317 | -0.5209 | No |
| 71 | CEP78 |  |  | 2000 | -0.317 | -0.5214 | No |
| 72 | RRP7A |  |  | 2017 | -0.319 | -0.5252 | No |
| 73 | PARPBP |  |  | 2026 | -0.322 | -0.5264 | No |
| 74 | KHSRP |  |  | 2027 | -0.322 | -0.5250 | No |
| 75 | TPGS2 |  |  | 2028 | -0.322 | -0.5236 | No |
| 76 | DDX39A |  |  | 2043 | -0.324 | -0.5267 | No |
| 77 | CNOT9 |  |  | 2047 | -0.325 | -0.5263 | No |
| 78 | SRRT |  |  | 2077 | -0.331 | -0.5342 | No |
| 79 | CCDC15 |  |  | 2080 | -0.331 | -0.5334 | No |
| 80 | E2F7 |  |  | 2081 | -0.331 | -0.5320 | No |
| 81 | CCNE1 |  |  | 2087 | -0.332 | -0.5322 | No |
| 82 | AHCY |  |  | 2089 | -0.333 | -0.5311 | No |
| 83 | ARPC5L |  |  | 2094 | -0.333 | -0.5309 | No |
| 84 | ZNF93 |  |  | 2095 | -0.333 | -0.5295 | No |
| 85 | CHEK1 |  |  | 2108 | -0.336 | -0.5319 | No |
| 86 | ABHD11 |  |  | 2109 | -0.336 | -0.5304 | No |
| 87 | ZNF367 |  |  | 2131 | -0.339 | -0.5358 | No |
| 88 | BRIX1 |  |  | 2135 | -0.340 | -0.5353 | No |
| 89 | MRM2 |  |  | 2145 | -0.342 | -0.5367 | No |
| 90 | TAF15 |  |  | 2146 | -0.342 | -0.5352 | No |
| 91 | DCXR |  |  | 2157 | -0.344 | -0.5369 | No |
| 92 | PHIP |  |  | 2162 | -0.345 | -0.5367 | No |
| 93 | PLK4 |  |  | 2167 | -0.346 | -0.5365 | No |
| 94 | COPS3 |  |  | 2175 | -0.348 | -0.5373 | No |
| 95 | YEATS4 |  |  | 2187 | -0.349 | -0.5393 | No |
| 96 | POC1A |  |  | 2199 | -0.351 | -0.5413 | No |
| 97 | MXD3 |  |  | 2201 | -0.352 | -0.5402 | No |
| 98 | TCOF1 |  |  | 2210 | -0.354 | -0.5412 | No |
| 99 | PKMYT1 |  |  | 2214 | -0.356 | -0.5406 | No |
| 100 | SMC1A |  |  | 2240 | -0.361 | -0.5471 | No |
| 101 | SAMD1 |  |  | 2245 | -0.362 | -0.5469 | No |
| 102 | FN3KRP |  |  | 2246 | -0.362 | -0.5453 | No |
| 103 | POLE |  |  | 2252 | -0.364 | -0.5453 | No |
| 104 | POLQ |  |  | 2253 | -0.364 | -0.5438 | No |
| 105 | PAFAH1B3 |  |  | 2264 | -0.365 | -0.5454 | No |
| 106 | PRPS1 |  |  | 2267 | -0.366 | -0.5445 | No |
| 107 | MTHFD1 |  |  | 2282 | -0.369 | -0.5474 | No |
| 108 | GLO1 |  |  | 2292 | -0.371 | -0.5487 | No |
| 109 | TIMM10 |  |  | 2296 | -0.372 | -0.5481 | No |
| 110 | RABL6 |  |  | 2301 | -0.373 | -0.5477 | No |
| 111 | DONSON |  |  | 2316 | -0.375 | -0.5506 | No |
| 112 | CKS1B |  |  | 2332 | -0.380 | -0.5538 | Yes |
| 113 | NOP56 |  |  | 2333 | -0.380 | -0.5522 | Yes |
| 114 | BUB1B |  |  | 2335 | -0.381 | -0.5509 | Yes |
| 115 | MCM2 |  |  | 2349 | -0.383 | -0.5534 | Yes |
| 116 | CCDC137 |  |  | 2352 | -0.384 | -0.5524 | Yes |
| 117 | NUP50 |  |  | 2354 | -0.385 | -0.5510 | Yes |
| 118 | CKAP2 |  |  | 2357 | -0.385 | -0.5500 | Yes |
| 119 | ZWILCH |  |  | 2358 | -0.385 | -0.5484 | Yes |
| 120 | MSH6 |  |  | 2362 | -0.386 | -0.5477 | Yes |
| 121 | PSIP1 |  |  | 2364 | -0.386 | -0.5463 | Yes |
| 122 | DKC1 |  |  | 2365 | -0.386 | -0.5447 | Yes |
| 123 | STIL |  |  | 2374 | -0.388 | -0.5456 | Yes |
| 124 | POLA1 |  |  | 2376 | -0.388 | -0.5442 | Yes |
| 125 | DDIAS |  |  | 2382 | -0.390 | -0.5441 | Yes |
| 126 | MEA1 |  |  | 2386 | -0.390 | -0.5434 | Yes |
| 127 | CENPW |  |  | 2402 | -0.393 | -0.5466 | Yes |
| 128 | UHRF1 |  |  | 2404 | -0.393 | -0.5452 | Yes |
| 129 | BID |  |  | 2405 | -0.393 | -0.5435 | Yes |
| 130 | PRIM2 |  |  | 2409 | -0.394 | -0.5428 | Yes |
| 131 | WDR34 |  |  | 2414 | -0.395 | -0.5423 | Yes |
| 132 | BUB1 |  |  | 2416 | -0.396 | -0.5409 | Yes |
| 133 | HAUS1 |  |  | 2423 | -0.397 | -0.5412 | Yes |
| 134 | WEE1 |  |  | 2426 | -0.397 | -0.5401 | Yes |
| 135 | HPF1 |  |  | 2435 | -0.399 | -0.5410 | Yes |
| 136 | ZNF738 |  |  | 2441 | -0.400 | -0.5408 | Yes |
| 137 | NCAPH2 |  |  | 2443 | -0.400 | -0.5394 | Yes |
| 138 | POLA2 |  |  | 2452 | -0.401 | -0.5403 | Yes |
| 139 | HPRT1 |  |  | 2454 | -0.402 | -0.5389 | Yes |
| 140 | CENPO |  |  | 2460 | -0.404 | -0.5387 | Yes |
| 141 | CKS2 |  |  | 2466 | -0.404 | -0.5386 | Yes |
| 142 | BLM |  |  | 2469 | -0.404 | -0.5375 | Yes |
| 143 | KIF11 |  |  | 2488 | -0.410 | -0.5415 | Yes |
| 144 | HAUS8 |  |  | 2494 | -0.412 | -0.5414 | Yes |
| 145 | CENPL |  |  | 2497 | -0.413 | -0.5402 | Yes |
| 146 | ECI1 |  |  | 2500 | -0.414 | -0.5391 | Yes |
| 147 | ZNF519 |  |  | 2504 | -0.416 | -0.5383 | Yes |
| 148 | POLR2D |  |  | 2510 | -0.416 | -0.5381 | Yes |
| 149 | CSE1L |  |  | 2512 | -0.417 | -0.5366 | Yes |
| 150 | C9orf40 |  |  | 2514 | -0.417 | -0.5351 | Yes |
| 151 | SNRPA |  |  | 2515 | -0.417 | -0.5333 | Yes |
| 152 | UBA2 |  |  | 2517 | -0.418 | -0.5318 | Yes |
| 153 | SPAG5 |  |  | 2519 | -0.418 | -0.5303 | Yes |
| 154 | RACGAP1 |  |  | 2522 | -0.419 | -0.5292 | Yes |
| 155 | NFATC2IP |  |  | 2531 | -0.423 | -0.5299 | Yes |
| 156 | CACYBP |  |  | 2536 | -0.424 | -0.5294 | Yes |
| 157 | PARP2 |  |  | 2542 | -0.425 | -0.5292 | Yes |
| 158 | PAICS |  |  | 2545 | -0.426 | -0.5280 | Yes |
| 159 | SLF1 |  |  | 2551 | -0.427 | -0.5277 | Yes |
| 160 | NAP1L4 |  |  | 2552 | -0.427 | -0.5259 | Yes |
| 161 | NCAPD2 |  |  | 2563 | -0.431 | -0.5273 | Yes |
| 162 | CDK5RAP2 |  |  | 2564 | -0.431 | -0.5254 | Yes |
| 163 | POLE3 |  |  | 2570 | -0.432 | -0.5251 | Yes |
| 164 | RSRC1 |  |  | 2571 | -0.433 | -0.5233 | Yes |
| 165 | LRR1 |  |  | 2579 | -0.434 | -0.5237 | Yes |
| 166 | SSRP1 |  |  | 2580 | -0.434 | -0.5218 | Yes |
| 167 | TMEM106C |  |  | 2592 | -0.437 | -0.5234 | Yes |
| 168 | C12orf75 |  |  | 2607 | -0.441 | -0.5260 | Yes |
| 169 | GGCT |  |  | 2610 | -0.442 | -0.5248 | Yes |
| 170 | KNTC1 |  |  | 2613 | -0.443 | -0.5235 | Yes |
| 171 | RMI1 |  |  | 2616 | -0.444 | -0.5222 | Yes |
| 172 | NCAPG2 |  |  | 2621 | -0.445 | -0.5216 | Yes |
| 173 | FANCB |  |  | 2623 | -0.446 | -0.5200 | Yes |
| 174 | PPIF |  |  | 2624 | -0.446 | -0.5181 | Yes |
| 175 | ANKRD9 |  |  | 2628 | -0.446 | -0.5171 | Yes |
| 176 | HIRIP3 |  |  | 2635 | -0.449 | -0.5171 | Yes |
| 177 | DCK |  |  | 2636 | -0.449 | -0.5152 | Yes |
| 178 | DCTPP1 |  |  | 2644 | -0.451 | -0.5155 | Yes |
| 179 | MASTL |  |  | 2646 | -0.452 | -0.5139 | Yes |
| 180 | EXOSC8 |  |  | 2659 | -0.456 | -0.5158 | Yes |
| 181 | PAQR4 |  |  | 2663 | -0.460 | -0.5147 | Yes |
| 182 | CCNA2 |  |  | 2667 | -0.462 | -0.5137 | Yes |
| 183 | RFWD3 |  |  | 2668 | -0.462 | -0.5117 | Yes |
| 184 | SKA2 |  |  | 2672 | -0.464 | -0.5107 | Yes |
| 185 | FIGNL1 |  |  | 2679 | -0.465 | -0.5106 | Yes |
| 186 | PGP |  |  | 2681 | -0.468 | -0.5089 | Yes |
| 187 | MND1 |  |  | 2682 | -0.468 | -0.5069 | Yes |
| 188 | KIF22 |  |  | 2683 | -0.468 | -0.5049 | Yes |
| 189 | CMC2 |  |  | 2694 | -0.470 | -0.5060 | Yes |
| 190 | CDCA8 |  |  | 2695 | -0.470 | -0.5040 | Yes |
| 191 | MMS22L |  |  | 2698 | -0.471 | -0.5026 | Yes |
| 192 | MCM6 |  |  | 2699 | -0.471 | -0.5006 | Yes |
| 193 | PPP1R14C |  |  | 2700 | -0.471 | -0.4986 | Yes |
| 194 | ANLN |  |  | 2702 | -0.472 | -0.4968 | Yes |
| 195 | HAT1 |  |  | 2703 | -0.472 | -0.4948 | Yes |
| 196 | PRC1 |  |  | 2705 | -0.472 | -0.4931 | Yes |
| 197 | FANCD2 |  |  | 2707 | -0.474 | -0.4914 | Yes |
| 198 | CENPN |  |  | 2708 | -0.474 | -0.4893 | Yes |
| 199 | MELK |  |  | 2709 | -0.474 | -0.4873 | Yes |
| 200 | SLC43A3 |  |  | 2713 | -0.475 | -0.4862 | Yes |
| 201 | TUBG1 |  |  | 2715 | -0.475 | -0.4845 | Yes |
| 202 | NDC80 |  |  | 2723 | -0.478 | -0.4846 | Yes |
| 203 | FAM136A |  |  | 2724 | -0.478 | -0.4826 | Yes |
| 204 | YWHAH |  |  | 2725 | -0.479 | -0.4805 | Yes |
| 205 | EXO1 |  |  | 2728 | -0.479 | -0.4791 | Yes |
| 206 | RPA3 |  |  | 2730 | -0.480 | -0.4773 | Yes |
| 207 | SHCBP1 |  |  | 2733 | -0.480 | -0.4759 | Yes |
| 208 | PIGX |  |  | 2736 | -0.482 | -0.4745 | Yes |
| 209 | BRIP1 |  |  | 2739 | -0.482 | -0.4730 | Yes |
| 210 | CHCHD3 |  |  | 2749 | -0.485 | -0.4738 | Yes |
| 211 | TIMELESS |  |  | 2752 | -0.487 | -0.4724 | Yes |
| 212 | UNG |  |  | 2758 | -0.489 | -0.4719 | Yes |
| 213 | CENPF |  |  | 2761 | -0.490 | -0.4704 | Yes |
| 214 | ACTL6A |  |  | 2762 | -0.492 | -0.4683 | Yes |
| 215 | THOP1 |  |  | 2763 | -0.492 | -0.4662 | Yes |
| 216 | IFRD2 |  |  | 2764 | -0.493 | -0.4640 | Yes |
| 217 | BRCA2 |  |  | 2772 | -0.495 | -0.4642 | Yes |
| 218 | NUF2 |  |  | 2781 | -0.497 | -0.4646 | Yes |
| 219 | SRSF2 |  |  | 2782 | -0.497 | -0.4624 | Yes |
| 220 | SLBP |  |  | 2792 | -0.499 | -0.4632 | Yes |
| 221 | MGME1 |  |  | 2797 | -0.500 | -0.4623 | Yes |
| 222 | DTYMK |  |  | 2798 | -0.501 | -0.4602 | Yes |
| 223 | WDR76 |  |  | 2800 | -0.501 | -0.4583 | Yes |
| 224 | RAD18 |  |  | 2802 | -0.501 | -0.4565 | Yes |
| 225 | FBL |  |  | 2804 | -0.502 | -0.4546 | Yes |
| 226 | RBL1 |  |  | 2808 | -0.503 | -0.4534 | Yes |
| 227 | ASF1B |  |  | 2824 | -0.510 | -0.4560 | Yes |
| 228 | CDCA3 |  |  | 2829 | -0.513 | -0.4551 | Yes |
| 229 | TMEM97 |  |  | 2834 | -0.514 | -0.4542 | Yes |
| 230 | SPC24 |  |  | 2836 | -0.514 | -0.4523 | Yes |
| 231 | HMGB3 |  |  | 2839 | -0.516 | -0.4507 | Yes |
| 232 | TOP2A |  |  | 2842 | -0.517 | -0.4491 | Yes |
| 233 | TOMM40 |  |  | 2845 | -0.519 | -0.4475 | Yes |
| 234 | E2F8 |  |  | 2847 | -0.519 | -0.4456 | Yes |
| 235 | LIG1 |  |  | 2848 | -0.520 | -0.4434 | Yes |
| 236 | CEP55 |  |  | 2852 | -0.522 | -0.4421 | Yes |
| 237 | WDHD1 |  |  | 2853 | -0.522 | -0.4398 | Yes |
| 238 | NABP2 |  |  | 2854 | -0.522 | -0.4376 | Yes |
| 239 | CKAP2L |  |  | 2857 | -0.523 | -0.4360 | Yes |
| 240 | ECT2 |  |  | 2858 | -0.523 | -0.4337 | Yes |
| 241 | NUDT1 |  |  | 2859 | -0.524 | -0.4314 | Yes |
| 242 | CD320 |  |  | 2860 | -0.525 | -0.4292 | Yes |
| 243 | PDLIM4 |  |  | 2861 | -0.525 | -0.4269 | Yes |
| 244 | RFC5 |  |  | 2862 | -0.526 | -0.4246 | Yes |
| 245 | CDK2 |  |  | 2863 | -0.526 | -0.4224 | Yes |
| 246 | TRIP13 |  |  | 2865 | -0.526 | -0.4204 | Yes |
| 247 | NUP62 |  |  | 2867 | -0.527 | -0.4185 | Yes |
| 248 | HJURP |  |  | 2869 | -0.527 | -0.4165 | Yes |
| 249 | POLR3K |  |  | 2872 | -0.528 | -0.4149 | Yes |
| 250 | PTTG1 |  |  | 2895 | -0.540 | -0.4196 | Yes |
| 251 | CDKN2C |  |  | 2903 | -0.544 | -0.4195 | Yes |
| 252 | PPM1G |  |  | 2907 | -0.545 | -0.4181 | Yes |
| 253 | MT2A |  |  | 2910 | -0.547 | -0.4164 | Yes |
| 254 | MRPL12 |  |  | 2911 | -0.547 | -0.4141 | Yes |
| 255 | FAM111A |  |  | 2917 | -0.552 | -0.4133 | Yes |
| 256 | NETO2 |  |  | 2920 | -0.556 | -0.4115 | Yes |
| 257 | ARHGAP11A |  |  | 2921 | -0.556 | -0.4091 | Yes |
| 258 | CCDC14 |  |  | 2929 | -0.560 | -0.4090 | Yes |
| 259 | CMSS1 |  |  | 2931 | -0.561 | -0.4069 | Yes |
| 260 | BRI3BP |  |  | 2932 | -0.562 | -0.4044 | Yes |
| 261 | BOLA3 |  |  | 2934 | -0.563 | -0.4023 | Yes |
| 262 | ATAD5 |  |  | 2935 | -0.563 | -0.3999 | Yes |
| 263 | FANCA |  |  | 2937 | -0.565 | -0.3978 | Yes |
| 264 | GMPS |  |  | 2939 | -0.566 | -0.3957 | Yes |
| 265 | H2AFY |  |  | 2942 | -0.568 | -0.3938 | Yes |
| 266 | CENPH |  |  | 2945 | -0.569 | -0.3920 | Yes |
| 267 | MIS18A |  |  | 2947 | -0.570 | -0.3899 | Yes |
| 268 | DSN1 |  |  | 2949 | -0.571 | -0.3878 | Yes |
| 269 | MAD2L2 |  |  | 2952 | -0.573 | -0.3859 | Yes |
| 270 | KIF4A |  |  | 2953 | -0.574 | -0.3834 | Yes |
| 271 | TACC3 |  |  | 2956 | -0.575 | -0.3816 | Yes |
| 272 | ACOT7 |  |  | 2957 | -0.576 | -0.3791 | Yes |
| 273 | CENPX |  |  | 2958 | -0.576 | -0.3766 | Yes |
| 274 | KIF23 |  |  | 2961 | -0.579 | -0.3748 | Yes |
| 275 | MZT1 |  |  | 2964 | -0.580 | -0.3729 | Yes |
| 276 | PIMREG |  |  | 2971 | -0.583 | -0.3723 | Yes |
| 277 | NCAPH |  |  | 2974 | -0.585 | -0.3704 | Yes |
| 278 | GGH |  |  | 2975 | -0.585 | -0.3679 | Yes |
| 279 | CTNNAL1 |  |  | 2976 | -0.585 | -0.3654 | Yes |
| 280 | POP7 |  |  | 2980 | -0.588 | -0.3638 | Yes |
| 281 | OLFM1 |  |  | 2981 | -0.589 | -0.3613 | Yes |
| 282 | VRK1 |  |  | 2983 | -0.592 | -0.3590 | Yes |
| 283 | SMC2 |  |  | 2984 | -0.592 | -0.3565 | Yes |
| 284 | SKA3 |  |  | 2985 | -0.592 | -0.3539 | Yes |
| 285 | KNL1 |  |  | 2986 | -0.592 | -0.3514 | Yes |
| 286 | PAXX |  |  | 2999 | -0.602 | -0.3526 | Yes |
| 287 | TPX2 |  |  | 3006 | -0.606 | -0.3520 | Yes |
| 288 | VMA21 |  |  | 3008 | -0.607 | -0.3497 | Yes |
| 289 | C19orf48 |  |  | 3013 | -0.608 | -0.3483 | Yes |
| 290 | TCF19 |  |  | 3019 | -0.613 | -0.3473 | Yes |
| 291 | RTKN2 |  |  | 3020 | -0.613 | -0.3446 | Yes |
| 292 | GINS4 |  |  | 3021 | -0.615 | -0.3420 | Yes |
| 293 | TFDP1 |  |  | 3027 | -0.617 | -0.3409 | Yes |
| 294 | TOPBP1 |  |  | 3033 | -0.621 | -0.3399 | Yes |
| 295 | DNMT1 |  |  | 3035 | -0.622 | -0.3375 | Yes |
| 296 | RBBP8 |  |  | 3053 | -0.637 | -0.3402 | Yes |
| 297 | AURKB |  |  | 3056 | -0.639 | -0.3381 | Yes |
| 298 | PHF19 |  |  | 3060 | -0.643 | -0.3363 | Yes |
| 299 | LMNB2 |  |  | 3061 | -0.644 | -0.3335 | Yes |
| 300 | JPT1 |  |  | 3062 | -0.644 | -0.3307 | Yes |
| 301 | NCAPD3 |  |  | 3064 | -0.646 | -0.3283 | Yes |
| 302 | ALYREF |  |  | 3068 | -0.649 | -0.3264 | Yes |
| 303 | SAC3D1 |  |  | 3073 | -0.655 | -0.3249 | Yes |
| 304 | FANCI |  |  | 3074 | -0.656 | -0.3221 | Yes |
| 305 | C21orf58 |  |  | 3078 | -0.659 | -0.3202 | Yes |
| 306 | CDC45 |  |  | 3081 | -0.661 | -0.3180 | Yes |
| 307 | GTSE1 |  |  | 3083 | -0.663 | -0.3154 | Yes |
| 308 | CDCA4 |  |  | 3084 | -0.663 | -0.3126 | Yes |
| 309 | TMPO |  |  | 3088 | -0.666 | -0.3107 | Yes |
| 310 | C1orf21 |  |  | 3094 | -0.669 | -0.3094 | Yes |
| 311 | MAD2L1 |  |  | 3099 | -0.675 | -0.3078 | Yes |
| 312 | NASP |  |  | 3106 | -0.682 | -0.3067 | Yes |
| 313 | RFC3 |  |  | 3108 | -0.683 | -0.3041 | Yes |
| 314 | KIFC1 |  |  | 3110 | -0.684 | -0.3015 | Yes |
| 315 | LSM4 |  |  | 3115 | -0.688 | -0.2998 | Yes |
| 316 | DBF4 |  |  | 3117 | -0.690 | -0.2971 | Yes |
| 317 | MYBL1 |  |  | 3119 | -0.691 | -0.2945 | Yes |
| 318 | LMNB1 |  |  | 3121 | -0.692 | -0.2918 | Yes |
| 319 | POLD3 |  |  | 3122 | -0.695 | -0.2888 | Yes |
| 320 | PRR11 |  |  | 3125 | -0.698 | -0.2864 | Yes |
| 321 | SMC4 |  |  | 3126 | -0.699 | -0.2834 | Yes |
| 322 | CENPK |  |  | 3127 | -0.700 | -0.2804 | Yes |
| 323 | RFC4 |  |  | 3129 | -0.701 | -0.2777 | Yes |
| 324 | MCM5 |  |  | 3132 | -0.706 | -0.2753 | Yes |
| 325 | GINS1 |  |  | 3137 | -0.713 | -0.2735 | Yes |
| 326 | GMNN |  |  | 3140 | -0.719 | -0.2710 | Yes |
| 327 | KIF15 |  |  | 3142 | -0.723 | -0.2682 | Yes |
| 328 | BTG3 |  |  | 3148 | -0.727 | -0.2667 | Yes |
| 329 | CDK1 |  |  | 3149 | -0.727 | -0.2636 | Yes |
| 330 | RNF168 |  |  | 3154 | -0.734 | -0.2617 | Yes |
| 331 | MKI67 |  |  | 3158 | -0.738 | -0.2595 | Yes |
| 332 | ORC6 |  |  | 3159 | -0.738 | -0.2563 | Yes |
| 333 | PRIM1 |  |  | 3160 | -0.739 | -0.2531 | Yes |
| 334 | TK1 |  |  | 3162 | -0.745 | -0.2502 | Yes |
| 335 | DNAJC9 |  |  | 3167 | -0.747 | -0.2483 | Yes |
| 336 | OXCT1 |  |  | 3176 | -0.756 | -0.2476 | Yes |
| 337 | MCM8 |  |  | 3178 | -0.758 | -0.2446 | Yes |
| 338 | CDT1 |  |  | 3179 | -0.758 | -0.2413 | Yes |
| 339 | MCM3 |  |  | 3182 | -0.761 | -0.2387 | Yes |
| 340 | USP1 |  |  | 3183 | -0.762 | -0.2354 | Yes |
| 341 | BRCA1 |  |  | 3185 | -0.765 | -0.2324 | Yes |
| 342 | PSMC3IP |  |  | 3194 | -0.772 | -0.2317 | Yes |
| 343 | XRCC2 |  |  | 3197 | -0.777 | -0.2290 | Yes |
| 344 | CIP2A |  |  | 3199 | -0.782 | -0.2259 | Yes |
| 345 | PXMP2 |  |  | 3200 | -0.782 | -0.2225 | Yes |
| 346 | BARD1 |  |  | 3201 | -0.783 | -0.2192 | Yes |
| 347 | SGO1 |  |  | 3202 | -0.783 | -0.2158 | Yes |
| 348 | CDCA7 |  |  | 3203 | -0.785 | -0.2124 | Yes |
| 349 | CDC6 |  |  | 3204 | -0.785 | -0.2090 | Yes |
| 350 | BIRC5 |  |  | 3208 | -0.793 | -0.2065 | Yes |
| 351 | FBXO5 |  |  | 3212 | -0.797 | -0.2041 | Yes |
| 352 | DIAPH3 |  |  | 3213 | -0.800 | -0.2006 | Yes |
| 353 | CARHSP1 |  |  | 3214 | -0.801 | -0.1971 | Yes |
| 354 | RRM1 |  |  | 3215 | -0.801 | -0.1937 | Yes |
| 355 | SPC25 |  |  | 3219 | -0.806 | -0.1912 | Yes |
| 356 | UBE2C |  |  | 3225 | -0.812 | -0.1893 | Yes |
| 357 | SYNE2 |  |  | 3231 | -0.820 | -0.1873 | Yes |
| 358 | NUSAP1 |  |  | 3237 | -0.825 | -0.1854 | Yes |
| 359 | FOXM1 |  |  | 3238 | -0.826 | -0.1818 | Yes |
| 360 | RFC2 |  |  | 3245 | -0.839 | -0.1801 | Yes |
| 361 | DTL |  |  | 3249 | -0.848 | -0.1774 | Yes |
| 362 | CENPM |  |  | 3269 | -0.881 | -0.1798 | Yes |
| 363 | CHAF1A |  |  | 3270 | -0.882 | -0.1760 | Yes |
| 364 | HUNK |  |  | 3272 | -0.884 | -0.1725 | Yes |
| 365 | CDCA5 |  |  | 3273 | -0.885 | -0.1686 | Yes |
| 366 | CCNE2 |  |  | 3276 | -0.889 | -0.1654 | Yes |
| 367 | FEN1 |  |  | 3278 | -0.890 | -0.1619 | Yes |
| 368 | NSD2 |  |  | 3281 | -0.896 | -0.1587 | Yes |
| 369 | H2AFX |  |  | 3282 | -0.898 | -0.1548 | Yes |
| 370 | HELLS |  |  | 3284 | -0.901 | -0.1512 | Yes |
| 371 | KLHL23 |  |  | 3287 | -0.907 | -0.1480 | Yes |
| 372 | E2F1 |  |  | 3289 | -0.911 | -0.1444 | Yes |
| 373 | CDKN3 |  |  | 3293 | -0.916 | -0.1414 | Yes |
| 374 | ZWINT |  |  | 3296 | -0.925 | -0.1380 | Yes |
| 375 | HMGA1 |  |  | 3299 | -0.931 | -0.1347 | Yes |
| 376 | TCEAL7 |  |  | 3306 | -0.939 | -0.1325 | Yes |
| 377 | FBLN1 |  |  | 3317 | -0.954 | -0.1316 | Yes |
| 378 | PCNA |  |  | 3319 | -0.963 | -0.1278 | Yes |
| 379 | UBE2T |  |  | 3330 | -0.979 | -0.1268 | Yes |
| 380 | ASRGL1 |  |  | 3333 | -0.984 | -0.1232 | Yes |
| 381 | EMP2 |  |  | 3338 | -0.998 | -0.1202 | Yes |
| 382 | MYBL2 |  |  | 3340 | -1.006 | -0.1161 | Yes |
| 383 | MCM4 |  |  | 3341 | -1.006 | -0.1118 | Yes |
| 384 | EZH2 |  |  | 3342 | -1.015 | -0.1074 | Yes |
| 385 | RAD51AP1 |  |  | 3345 | -1.030 | -0.1036 | Yes |
| 386 | PBK |  |  | 3354 | -1.046 | -0.1017 | Yes |
| 387 | MCM10 |  |  | 3356 | -1.050 | -0.0975 | Yes |
| 388 | ESCO2 |  |  | 3367 | -1.088 | -0.0960 | Yes |
| 389 | NCAPG |  |  | 3370 | -1.098 | -0.0919 | Yes |
| 390 | MCM7 |  |  | 3384 | -1.147 | -0.0911 | Yes |
| 391 | GINS2 |  |  | 3386 | -1.156 | -0.0865 | Yes |
| 392 | DHFR |  |  | 3387 | -1.159 | -0.0815 | Yes |
| 393 | HMGB2 |  |  | 3390 | -1.184 | -0.0770 | Yes |
| 394 | CLSPN |  |  | 3391 | -1.184 | -0.0719 | Yes |
| 395 | UBE2S |  |  | 3396 | -1.205 | -0.0680 | Yes |
| 396 | CENPU |  |  | 3398 | -1.230 | -0.0630 | Yes |
| 397 | HIST1H1E |  |  | 3401 | -1.237 | -0.0583 | Yes |
| 398 | FAM111B |  |  | 3402 | -1.238 | -0.0529 | Yes |
| 399 | MCUB |  |  | 3405 | -1.245 | -0.0482 | Yes |
| 400 | PCLAF |  |  | 3409 | -1.265 | -0.0437 | Yes |
| 401 | ATAD2 |  |  | 3415 | -1.278 | -0.0398 | Yes |
| 402 | RRM2 |  |  | 3420 | -1.297 | -0.0355 | Yes |
| 403 | HES6 |  |  | 3431 | -1.366 | -0.0328 | Yes |
| 404 | TYMS |  |  | 3436 | -1.391 | -0.0281 | Yes |
| 405 | DRAXIN |  |  | 3443 | -1.493 | -0.0236 | Yes |
| 406 | HIST1H1D |  |  | 3466 | -1.743 | -0.0231 | Yes |
| 407 | HIST1H1B |  |  | 3467 | -1.770 | -0.0155 | Yes |
| 408 | CDCA7L |  |  | 3481 | -1.878 | -0.0116 | Yes |
| 409 | HIST1H4C |  |  | 3493 | -2.248 | -0.0054 | Yes |
| 410 | PMP2 |  |  | 3498 | -2.520 | 0.0042 | Yes |
Table: GSEA details [plain text format]

  

Fig 2: DOROSZKO\_U3054MG\_CLUSTER\_4: Random ES distribution      
 Gene set null distribution of ES for **DOROSZKO\_U3054MG\_CLUSTER\_4**
